# Exploration of chromene-based BioAIEgens

**DOI:** 10.1093/nsr/nwae064

**Published:** 2024-02-26

**Authors:** Fritz E Kühn

**Affiliations:** Department of Chemistry & Catalysis Research Center, School of Natural Sciences, Technical University of Munich, Germany

Photoemission efficiency is usually higher in solution than in solid state. Some compounds however, display a reverse behavior. Such a behavior is called aggregation-induced emission (AIE). Synthetic AIE luminogens (AIEgens) have gained significant interest during the past two decades. They can be applied, for instance, as sensitive light-up probes, allowing non-invasive tracking *in vivo* in real time. The exploration of AIE properties originating from natural products, known as BioAIE, is a highly promising approach, for biocompatibility is a very important issue for medical applications particularly, but not only in medicine. However, the number of accessible and applicable BioAIEgens is still rather limited.

Xu-Min Cai and Shenlin Huang from Nanjing Forestry University, as well as Ben Zhong Tang and Zheng Zhao from The Chinese University of Hong Kong-Shenzhen, modify natural structural scaffolds in order to broaden the scope of available BioAIEgens and to present an alternative to the solely extraction-based approach to novel systems. Recently they reported a series of BioAIEgens containing a natural chromene scaffold [1]. Cai's and Tang's groups have examined BioAIE materials, such as rosin [2], coumarin [3,4], and lignin [5]. The natural chromene scaffold serves as a structural analogue to commonly used dyes such as coumarin and chromone, and is also a fundamental component of numerous natural drugs (Acetovanillochromene) and commercial dyes (rhodamine and fluorescein). Compared to coumarin and chromone, chromene contains a non-conjugated sp^3^ carbon, which Cai *et al.* consider as similar to the alkyl carbon of a rosin alicyclic ring, and assume it may have a special impact on luminescent properties, making it a worthwhile target to study. To cope with the fact that the number of natural compounds containing chromene scaffolds is low and often difficult to extract in large enough quantities, the authors applied synthetic methodologies to design molecules containing chromene scaffolds, and explored their luminescence mechanisms, structure-property relationships, and application values.

**Figure 1.**
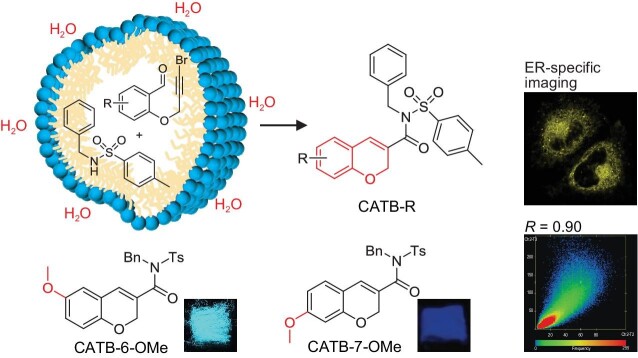
Synthesis in water is conducted to produce chromene-based BioAIEgens with regiostructure-, polymorphism- and substituent-dependent fluorescence and ER-specific imaging (Adapted from [1]).

Instead of applying organic solvents, the authors opted for an aqueous micellar system, utilizing an environmentally friendly, sustainable pathway and obtained a series of chromene-based derivatives with AIE properties (Fig. 1). The products exhibit regiostructure-dependent fluorescence in both solution and solid state, showing that 6-substitution displays a stronger red-shift emission than 7-substitution, indicating that the molecular and aggregation properties can be effectively adjusted through positional isomer design at a molecular level. Furthermore, the modified behavior of aggregated structures in comparison to the behavior of the consisting single molecules becomes most significant at the macroscopic level. In specific aggregated structures, the sum and synergy of various effects can promote the expansion and transformation of a systemic performance, achieving functionalities that surpass the intrinsic molecular properties. The concept of ‘Molecular Uniting Set Identified Characteristic (MUSIC)’ proposed by Li *et al*. [6] also emphasizes the core role of molecular aggregated states. Here, the mechanochromic properties of CATB-6-OMe and CATB-6-Me, as well as the polymorphism-dependent fluorescence of CATB-6-OMe, also demonstrate the important role of aggregated structures with respect to their macroscopic optical properties. The article exemplarily elucidates structure-property relationships between macroscopic optical properties and molecular structures as well as aggregated structures. Finally, the chromene derivatives were applied in cellular imaging, confirming the feasibility of using chromene derivatives for ER-specific imaging. Cell experiments also proved that tunable fluorescence based on tailor-made ligand modifications can modulate the cell vitality and imaging behavior of such BioAIEgens, providing more flexible applications.

The authors impressively demonstrated the application of a new strategy towards modified BioAIEgens by using an aqueous phase synthetic method to straightforwardly introduce useful variations into structures. This approach opens creative pathways for the development of novel BioAIEgens.
